# Physical Fitness Profiles of Young Female Team Sport Athletes from Portuguese Rural Settings: A Cross-Sectional Study

**DOI:** 10.3390/sports13080248

**Published:** 2025-07-28

**Authors:** Bebiana Sabino, Margarida Gomes, Ana Rodrigues, Pedro Bento, Nuno Loureiro

**Affiliations:** 1 Higher School of Education, Polytechnic Institute of Beja, 7800-000 Beja, Portugal; margarida.gomes@ipbeja.pt (M.G.); pbento@ipbeja.pt (P.B.); nloureiro@ipbeja.pt (N.L.); 2 SPRINT, Sport Physical Activity and Health Research & Innovation Center, 7800-000 Beja, Portugal; 3 Department of Physical Education and Sport, Faculty of Social Sciences, University of Madeira, 9020-105 Funchal, Portugal; anajar@staff.uma.pt; 4 Research Center in Sports Sciences, Health Sciences, and Human Development (CIDESD), 5001-801 Vila Real, Portugal; 5 Centre for Tourism Research, Development, and Innovation (CITUR), Madeira, 9020-105 Funchal-Madeira, Portugal

**Keywords:** girls, adolescents, handball, volleyball, basketball, football, anthropometry, agility, sprint, strength

## Abstract

**Background:** Sports performance indicators are mainly based on male athletes, highlighting the importance of portraying the female reality, particularly in rural contexts. This study aims to characterize sports performance indicators (body composition and physical fitness) of young Portuguese female athletes. **Methods:** A cross-sectional study was conducted with 124 girls (13.66 ± 1.93 years) participating in federated team sports in a rural region of Portugal. Body composition was assessed using bioelectrical impedance, and physical fitness was evaluated through vertical jump tests (countermovement jump and squat jump), sprint (20 m), agility (*T*-test), handgrip strength, and cardiovascular endurance (Yo-Yo IR1). **Results:** Volleyball players are taller; football and basketball players are heavier; football and volleyball players have more fat-free mass than handball players (*p* < 0.05). Body mass index and % body fat did not differ between sports (*p* > 0.05). Volleyball players performed better in the countermovement jump (F = 4.146, *p* = 0.008) and squat jump (F = 7.686, *p* < 0.001) when compared to basketball, football, and handball players. No differences were observed in the speed or cardiorespiratory endurance tests (*p* > 0.05). **Conclusions:** The results revealed that, despite some specific differences between sports, most physical fitness indicators did not differ significantly between sports after controlling for age, menarche, and training experience. These findings suggest that shared contextual limitations in rural regions may take precedence over sport-specific adaptations in the early stages of sports participation.

## 1. Introduction

Historically, women and girls have experienced limited opportunities for participation in sports compared to boys and men, and there has traditionally been, and remains, vast gender inequality in sport [1,2]. For example, sports media generally dedicates only 5% to 8% of coverage to women’s sports, even though 40% of sports participation is by women [3]. Although women have faced barriers in competing and being recognized, they have gradually gained more space and opportunities. Women’s participation rates in the Olympic Games have been gradually increasing, with the number of male and female athletes reaching parity for the Paris 2024 Olympic Games [4]. A recent study shows that between 2016 and 2018, there was a significant increase in female participation in traditionally male-dominated sports [5]. A similar trend with similar growth patterns is evident in other countries, such as Portugal, where the number of registered female athletes has increased significantly over the past two decades. In 2003, there were 70,051 female athletes, which increased to 133,471 in 2013 and reached 243,917 in 2023 [6]. The increasing participation in sports can reap additional benefits beyond those associated with performance, such as a reduction in stress, anxiety, and depression; improved social, emotional, and behavioral skills; enhanced self-esteem; and a decrease in obesity [7].

Despite recent progress, female participation in organized sports in Portugal is still much lower than male participation. This fact highlights structural gaps in girls’ and women’s access, retention, and appreciation for sports practice. This inequality is even more obvious in outlying and rural areas, such as Alentejo, where resources, infrastructure, and chances for women’s sports are scarce [6].

Throughout life, from childhood to adulthood, participation in sports is essential for a positive start to performance and, consequently, for progress in developing specific skills and physical attributes. Among these stages, adolescence is the phase of physiological and psychological growth and development [8], which for women includes the onset of the menstrual cycle, having a direct impact on overall performance [9,10]. Puberty also brings changes for girls, such as a higher percentage of body fat than men and less muscle mass for a given body size, and they also develop larger breasts and wider hips [11]. In addition to musculoskeletal changes and variations in strength development, hormonal changes, such as increased estrogen levels, also affect the properties of muscles and ligaments, particularly the anterior cruciate ligament [12]. These physiological changes in girls after puberty have an impact on their sporting performance, with boys generally outperforming girls in most fitness components, including speed, strength, power, and endurance [13].

Given these physiological and biomechanical variations, several aspects of exercise prescription may require sex-specific recommendations [14]. However, women continue to be underrepresented in sports research, underscoring the need for in-depth studies that can inform the development of training methods tailored to their specific needs [15,16]. Consequently, due to the historical predominance of research and athletic participation among men compared to women [15], there is a lack of knowledge about the physiology of women athletes, their athletic ability, and the acute and adaptive responses of women to exercise and training relative to men. This gap is particularly evident in young female athletes in Portugal, where research is scarce [17]. Notably, understanding these factors is crucial for developing tailored training programs, contributing to the literature on female youth sports, and informing regional sports development with inclusive, evidence-based approaches. A significant step toward closing the knowledge gap is to include a more and an equitable number of women in mechanistic studies that determine any sex differences in response to an acute bout of exercise, exercise training, and athletic performance.

Although the literature indicates an increase in girls’ and women’s participation in sports, the gap with men’s sports remains, especially in less developed areas where prejudice persists and the range of opportunities is still limited [18]. Women’s participation in team sports in rural areas continues to face significant challenges. Economic barriers, the lack of sporting culture and infrastructure, the absence of career opportunities, and sociocultural constraints all limit women’s involvement in sport, and these factors are particularly acute in rural contexts [19]. The geographic isolation of rural areas exacerbates these difficulties, as registration, equipment, and transport costs are higher than for those living in urban settings [20]. The social and cultural landscape of rural communities adds yet another layer of complexity to women’s participation in sports. Traditional gender roles and social expectations may be more deeply entrenched in these regions, where certain parental attitudes and gender constraints still influence whether families prioritize sport for their daughters [20,21].

Therefore, this study aims to contribute knowledge about athletes who face dual challenges—being women and living in a rural area—where the first girls’ team sports are only now emerging. The purpose of this study was to characterize and compare indicators of body composition and physical fitness in adolescent girls participating in different team sports (handball, basketball, football, and volleyball), as well as to investigate the impact of age, menarche, and time spent practicing sports on these variables.

## 2. Materials and Methods

### 2.1. Study Sample and Design

A total of 124 girls aged between 10 and 17.9 years (13.66 ± 1.93 years) took part in the study. The participants were recruited using a convenience sampling method, involving all federated clubs in the district of Beja (Portugal) that had teams in one of the four team sports considered: handball (*n* = 35), football (*n* = 42), basketball (*n* = 25), and volleyball (*n* = 22). Although there was no predetermined sample size calculation, all eligible clubs in the district were invited to participate in the study. Of the seven clubs contacted, six agreed to participate, providing comprehensive representation of the regional female athlete population.

The inclusion criteria were the following: (i) female athletes aged between 10 and 17.9 years old; (ii) federated athletes with at least one full competitive season in their sport; (iii) not being in any treatment for an injury; and (iv) had written informed consent provided by both the participant and her legal guardian. The exclusion criteria were the following: (i) less than one full season of federated sports practice; and (ii) presence of any diagnosed health condition or injury at the time of data collection.

The girls trained in club-based teams, with clubs originating from different rural towns within the Beja district, resulting in geographically diverse sample. Training frequency ranged from 2 to 3 sessions per week, with an average session duration of 90.

The participants and their legal guardians authorized participation in the study through a process of informed consent. The tutors and participants were introduced to the study, its objectives and assessment protocols, and the team was available to clarify any doubts. The study complies with the Declaration of Helsinki and has been approved by the Ethics Committee of Polytechnic University of Beja, with reference 1/2020. The confidentiality and anonymity of the information collected were safeguarded, with the information used only by members of the research team and personal data coded.

All assessments were carried out between January and May 2024, during one of the athletes’ regular weekly training sessions, as specified by each club. The evaluations were conducted on a single day, in a standardized order for all participants. The sequence of tests was as follows: (i) body composition assessment; (ii) strength tests: handgrip strength, countermovement jump (CMJ), and squat jump (SJ); (iii) agility and speed: *T*-test and 20 m sprint; and (iv) cardiorespiratory fitness: Yo-Yo Intermittent Recovery Test Level 1 (Yo-Yo IR1). Data collection was conducted by the researchers, who are the authors of this study. Before each test was carried out, a member of the research team explained the procedures.

### 2.2. Measures

#### 2.2.1. Body Composition

Body height measurement was performed with a Seca anthropometer (SECA, Hambourgh, Germany) (measurement accuracy 0.1 cm).

A bioelectrical impedance analyzer (Tanita^®^ BC-601, Tokyo, Japan) was used to study body composition. The participants remained barefoot on the two metal electrodes of the measurement platform, without socks or stockings, and with clean soles of their feet, refraining from eating or exercising for approximately three hours. Parameters measured during the assessment included body fat percentage, fat-free mass, body mass, and body mass index. This instrument has shown acceptable levels of reliability and validity for assessing body composition in adolescent populations [22].

#### 2.2.2. Physical Fitness Tests

The SJ and CMJ variables were measured with photoelectronic cells (Optojump Next, Microgate, Italy), as described by Glatthorn et al. [23]. Jump heights were calculated using the recorded contact and flight times of vertical jumps at a frequency of 1 kHz. For every test, the best three-jump performance was noted, and a 30-second recuperation followed each trial. For the CMJ, participants began in an upright standing position with their hands placed on their hips throughout the movement to eliminate any influence of arm swing. Each participant performed a rapid downward movement until the knees reached approximately 90 degrees of flexion, followed immediately by a vertical jump as high as possible, landing on both feet simultaneously. Participants also began the SJ in an upright standing position with their hands on their hips before transitioning into a static squat position with their knees bent at around a 90-degree angle. They performed a maximum vertical jump, landing on both feet once more, after maintaining the position for around two seconds without making any preemptive countermovement.

Handgrip strength was assessed with a handgrip test using a dynamometer (Jamar, Irvington, NY, USA). Athletes were instructed to perform the test in a standing position, with the shoulder adducted and neutrally rotated, the elbow flexed at 90 degrees, the forearm in a neutral position, and the wrist between 0 and 30 degrees of extension. Each participant performed three maximal isometric contractions using the dominant hand, with a 30 s rest between trials to prevent fatigue. The best result obtained (in kilograms) was recorded and used for analysis.

The *T*-test was employed to assess agility performance, following the standardized protocol described by Castillo et al. [24]. The test consisted of a predefined T-shaped running course, with cones placed at four specific points to delineate the circuit: one at the starting point, one 10 m straight ahead, and two others placed 5 m laterally to the left and right of the center cone, forming the top of the “T”. A photoelectric timing system (Microgate Polifemo Radio Light) was used, with the sensor beam positioned at the start/finish line to ensure accurate time measurement. Each athlete began the test from a standing position, 0.2 m behind the start beam, and performed the circuit at maximum effort. The course required the participant to: (i) sprint forward to the middle cone (10 m); (ii) side-shuffle to the left cone (5 m); (iii) side-shuffle to the far-right cone (10 m total across the top of the “T”); (iv) return to the middle cone (5 m); and (v) backpedal to the start line (10 m). The height of the photoelectric cells was individually adjusted to the athlete’s hip level to ensure consistent detection. Each athlete performed two valid attempts, with a minimum rest interval of 3 min between trials to allow for full recovery. The fastest time was recorded and used for analysis.

For the sprint test, athletes were instructed to stand in an upright position, 0.2 m behind the starting photoelectric beam, and to cover the 20 m distance as fast as possible. The time, recorded in seconds, was measured using a photoelectric cell system (Microgate Polifemo Radio Light). The height of the photocells was adjusted individually to match each participant’s hip height, ensuring consistent beam alignment. Each athlete had two attempts, with a minimum recovery time of 6 min between each, and the best time was used in the analysis.

The Yo-Yo IR1 was administered to evaluate performance in intermittent endurance [25]. The test involved 20 m shuttle runs at increasing speeds until exhaustion, with 10 s active recovery intervals (2 m × 5 m of jogging) between each run. The test ended when the participant felt incapable of completing another shuttle at the required pace (subjective judgment) or failed to reach the front line in time on two consecutive occasions (objective evaluation). The test score was determined by the number of runs made. At least one trial was used to acquaint the subjects with the test. The Yo-Yo IR1’s reliability has been demonstrated with an ICC of 0.94 and a CV of 3.6% [25].

### 2.3. Statistical Analysis

Data processing and analysis were conducted using IBM SPSS Statistics version 28.0 (SPSS Inc., Chicago, IL, USA). The level of significance adopted was 5%.

Descriptive statistics, including the mean and standard deviation, were used to characterize the sample. The chi-square test was used to determine the independence between the occurrence or non-occurrence of menarche and the practice of different sports. The normality of variable distributions was verified through the Shapiro–Wilk test, while homogeneity of variances was assessed using Levene’s test.

To examine the multivariate effect of sports on body composition and physical fitness, three distinct statistical models were employed. The first model consisted of a Multivariate Analysis of Variance without covariates. Subsequent models utilized Multivariate Analysis of Covariance, with the second model adjusted for age and menarche as covariates, and the third model additionally adjusted for years of sports practice.

The fundamental assumptions for Multivariate Analysis of Covariance application were rigorously verified prior to analysis. Multivariate normality was assessed through univariate Shapiro–Wilk tests and visual inspection of residual plots. Homogeneity of covariance matrices was tested using Box’s M test, ensuring the validity of the obtained results. Post hoc analyses were performed using the Bonferroni correction to identify pairwise differences between sports.

## 3. Results

The sample consists only of girls who, on average, have an age of 13.66 ± 1.93 years, and practice the following sports: (i) handball (28.2%), (ii) basketball (20.2%), (iii) football (33.9%), or (iv) volleyball (17.7%). Most of them have a sports past (83.9%) and report menarche (75.8%), which occurred on average at 11.41 ± 1.21 years of age. The characterization of the sample, as evaluated by the indicators of body composition and physical fitness, is reported in Table 1.

### 3.1. Body Composition

Handball players were, on average, the youngest and had the lowest proportion of athletes who had experienced menarche. They were also significantly shorter, lighter, and had lower fat-free mass compared to athletes from the other sports (*p* < 0.05). Volleyball players report a shorter time spent on sports practice, while basketball players report a longer time (*p* < 0.05) (Table 2). Although there was no significant difference in body mass index or body fat percentage between sports, the observed disparities in anthropometric measures, notably among handball players, could be attributed to both sport-specific traits and age-related developmental differences.

After adjusting for age, menarche occurrence, and time spent playing sports, the multivariate analysis indicated no statistically significant differences across sports in any of the body composition variables examined (Table 3). As a result, the variations in Table 2 are explained mainly by maturational characteristics and sporting experience, rather than the sport itself.

### 3.2. Physical Fitness

A multivariate analysis of variance revealed statistically significant differences in the overall physical fitness profile among athletes from different sports (*Pillai’s* trace = 0.622, F_(18,303)_ = 4.400, *p* < 0.001, *η*^2^ = 0.207). Univariate analyses indicated significant differences between sports in CMJ (F_(3,120)_ = 4.474, *p* = 0.005, *η*^2^ = 0.114), SJ (F_(3,120)_ = 8.880, *p* < 0.001, *η*^2^ = 204), and handgrip strength (F_(3,120)_ = 4.198, *p* = 0.008, *η*^2^ = 0.108). Volleyball players showed significantly higher performance in both vertical jump tests (CMJ and SJ) compared to players from handball, basketball, and football. Regarding handgrip strength, basketball players presented higher values on average than handball players. No significant differences were found between sports in the YO-YO IR1 (Table 4).

After controlling for age and menarche, statistically significant differences were found between sports in physical fitness (*Pillai’s* trace = 0.586, F_(18,339)_ = 4.008, *p* < 0.001, *η*^2^ = 0.195). Univariate analyses revealed significant differences in agility (F_(18,124)_ = 3.184, *p* < 0.027, *η*^2^ = 0.086), CMJ (F_(18,124)_ = 3.992, *p* = 0.01, *η*^2^ = 0.105), SJ (F_(18,124)_ = 7.880, *p* < 0.001, *η*^2^ = 0.188), and handgrip strength (F_(18,124)_ = 4.031, *p* = 0.009, *η*^2^ = 0.106). Volleyball players showed higher performance in the CMJ and SJ tests compared to handball, basketball, and football players. Basketball players outperformed handball and volleyball players in handgrip strength. However, after adjusting for age, menarche, and years of sports practice, the differences in handgrip strength and agility were no longer statistically significant (*p* = 0.080 and *p* = 0.102, respectively), suggesting that training experience may account for part of the previously observed differences. Only the CMJ (F_(18,124)_ = 4.146, *p* = 0.008, *η*^2^ = 0.110) and SJ (F_(18,124)_ = 7.686, *p* < 0.001, *η*^2^ = 0.186) tests showed statistically significant differences across groups, with volleyball players still outperforming basketball, football, and handball players (Table 5).

## 4. Discussion

This is one of the first studies to examine body composition and physical fitness in young female team sports athletes from a rural region in Portugal, considering age, menarche, and years of training. The main objective of this study was to characterize body composition and various indicators of motor performance, and to determine if there are differences between sports, considering age, years of practice, and the onset of menarche.

### 4.1. Body Composition

Body composition in young female athletes participating in team sports is a multifaceted topic influenced by various factors, including the type of sport, training regimens, and sociocultural dynamics.

Handball players are the shortest, lightest, and have a lower body fat percentage and lower fat-free mass compared to athletes from other sports. However, their anthropometric measurements are also lower compared to those of other female handball players, as seen in a study conducted with Hungarian teenagers (average age 14.2 years) [26]. In both cases, this may be related to the fact that in our study, the handball players were the youngest. However, even when compared to the reference values of Tunisian female athletes aged 13 [27], the athletes in our study are shorter, lighter, have lower fat-free mass, and a higher body fat percentage. The profile of female athletes shows that handball players are significantly shorter than basketball players and have a higher body fat percentage than football players [28].

In our study, the variables weight and body fat percentage did not differ between the various sports under analysis. However, in the height and fat-free mass variables, the volleyball players showed higher values than the handball players, as observed in the study conducted by Joksimovic et al. [29] on young handball and volleyball players. Football players also showed higher values than handball players for fat-free mass, in our study, as was also observed by Masanovic [30].

Despite the findings of our study, the literature shows that body fat percentage and fat-free mass differ between athletes from various sports. Football players exhibit lower lean mass metrics compared to volleyball and handball players, indicating a distinct body composition profile [31]. Volleyball players tend to have higher fat mass compared to athletes in football [32]. Female athletes in team sports generally exhibit higher fat-free mass and skeletal muscle mass compared to those in endurance sports [33].

The distinctions between the various sports disappear when considering the body composition factors, which account for factors such as age, playing time, and menarche. These findings lead us to believe that the typical body composition profile for each sport has not yet been established, as all athletes are still in the early stages of practice, regardless of the team sport they participate in. The literature indicated that training, particularly fitness training, has a significant impact on the body composition of young athletes [26,34]. This specificity of training comes from the time spent practicing.

Studies on athletes have shown that menarche status influences changes in body composition, specifically body mass index, body fat percentage, and fat-free mass [35]. This may explain why, after controlling for this variable, differences between sports were no longer statistically significant. Although not assessed in the present study, previous research suggests that the use of oral contraceptives and hormonal fluctuations may also influence body composition in female athletes [36]. Although studies indicate that their use does not significantly alter body composition parameters, a slight increase in body fat percentage is observed among female users [37]. These aspects help contextualize the findings and should be taken into consideration in future studies.

### 4.2. Physical Fitness

Physical fitness in young female sport team athletes is a comprehensive field of study that encompasses strength, agility, cardiorespiratory endurance, and other components. Research indicates that specialized training programs tailored to the demands of various sports can enhance physical fitness levels. In the particular instance of our research, it was expected that, regardless of the sport, the athletes’ performance in the physical fitness tests would be worse than that of athletes of the same age due to the short amount of time they had been practicing and the absence of female sports culture in team sports in this region of the country. However, this was not confirmed in all the sports and components of physical fitness. In handball, Portuguese players in this study had lower values than Romanian athletes in SJ and CMJ [38] and Tunisian athletes in the *T*-test [39]. Additionally, in the 20 m sprint test, the scores were lower than those of other under-14 athletes, although the handgrip test results are similar [40]. In basketball, the athletes in this study performed worse than elite Portuguese athletes of similar ages in terms of speed, agility, and handgrip strength. Consequently, they performed better in the CMJ [41]. In this study, volleyball players outperformed their peers in the same age group in terms of speed in the 20 m sprint and CMJ [42]. Football players in this study performed equally in the CMJ to non-elite athletes but were less successful in the YO-YO test [43]. Comparisons with elite athletes were used to contextualize performance levels; in this regard, average performance in lower limb strength (SJ and CMJ) and upper limb strength (handgrip strength) was similar to that observed in elite female athletes [44].

In our study, no differences were found between the various sports in terms of speed, agility, and cardiorespiratory fitness tests. However, after controlling for age and menarche, volleyball players showed significantly better agility compared to handball players. These differences, however, were no longer significant when the duration of sports practice was also included as a covariate, suggesting that years of training may play a more relevant role in agility development than sport modality alone. Previous research, particularly involving male athletes, has reported better agility in volleyball players, while football players tend to show lower correlations between speed and agility performance [45]. However, in the upper and lower limb strength tests, differences are observed between sports in this study. In the CMJ and Squat Jump, the volleyball players showed superior performance, as seen in other studies comparing the jumping performance of young athletes in the team sports under analysis [46,47]. These findings underscore the importance of tailoring training and development strategies to the unique physical demands of each sport. For example, volleyball players’ greater jumping performance could reflect both the sport’s emphasis on vertical power and the necessity for specialized plyometric and strength training. Recognizing these sport-specific profiles may help coaches and physical educators develop more effective and developmentally appropriate training programs, potentially enhancing athletic development and reducing the risk of injury in young female athletes.

Successful athletic performance is influenced by each of the components of physical fitness. However, since we are considering young athletes, the analysis of these variables must not forget the process of growth and development, which causes neurological, structural, and metabolic changes in the musculoskeletal system [48]. This is why, in our study, we used chronological age and the onset of menarche as indicators of maturation. In addition, the impact of specific training should also be considered when analyzing these variables, such as the effect of plyometric and strength training on the jumping ability of young athletes [49]. Thus, the results achieved by the volleyball players in our study, compared to those in other sports, can also be explained by the fact that they are older, more mature, and have been practicing the sport for longer.

Overall, body composition and specific physical fitness tests can be associated with injury risk, making it possible to identify physical abnormalities that can aid in prevention, particularly in knee injuries characteristic of young female athletes [50,51].

One of the study’s limitations is its cross-sectional design, which prevents causal inferences from being drawn from the observed associations. Furthermore, the same test battery was used across all sports, which, although beneficial for comparative reasons, may have overlooked sport-specific demands, thereby limiting the ecological validity of specific assessments. Another limitation of the study is that it does not analyze specific positions in each sport, as both body composition and physical fitness seem to vary according to the positions occupied on the pitch [36,52]. Additionally, the relatively small sample size, mainly when divided across four sports, may limit the generalizability and statistical power of the findings. However, this reflects the actual number of eligible female athletes in team sports within the rural region under study. Future studies should consider expanding data collection to neighboring districts or employing multi-center designs to increase sample size and representativeness. Moreover, the time of the season in which the assessment takes place should be considered, since changes in body composition occur throughout the season [53]. To facilitate comparisons between sports, the same tests were used for all sports, which sometimes suggest that the test used to assess a specific component of physical fitness may not be the most suitable for that sport.

The results of this study enabled the characterization of the body composition and physical fitness profiles of young female athletes participating in team sports for the first time in a rural region. Despite some sport-specific differences, such as superior jumping performance in volleyball players and greater upper-limb strength in basketball players, most physical fitness indicators did not differ significantly between sports after controlling for age, menarche, and training experience. These results suggest that shared contextual constraints (such as facility availability, specialized coaching, recent history of the sport’s implementation in the region, or training volume) may take precedence over sport-specific adaptations in the early phases of sport participation. In practical terms, these findings underscore the importance of funding inclusive, long-term development initiatives that take account for the specific challenge of remote areas. To compensate for the lack of early sport specialization, coaches in rural areas should emphasize the fundamentals of physical fitness and provide a variety of training stimuli. Targeting fair access to resources and coaching knowledge across sports could also help local sports policy, allowing for a more balanced athletic development and retention of female players in rural regions.

## Figures and Tables

**Table 1:** Sample characteristics (n = 124).

	Total
Age (years) (mean ± standard deviation)	13.66 ± 1.93
Sport *n* (%)	
Handball	35 (28.2%)
Basketball	25 (20.2%)
Football	42 (33.9%)
Volleyball	22 (17.7%)
Sporting past in another sport *n* (%)	
Yes	104 (83.9%)
No	20 (16.1%)
Menarche n (%)	
Yes	94 (75.8%)
No	30 (24.2%)
Age of Menarche (mean ± sd)	11.41 ± 1.21
Body composition (mean ± sd)	
Height (cm)	156.20 ± 9.29
Weight (kg)	51.87 ± 12.4
BMI ^1^ (kg/m^2^)	21.08 ± 3.99
Body Fat (%)	13.24 ± 7.12
Water (Kg)	28.29 ± 4.68
Fat-Free Mass (kg)	38.65 ± 6.41
Physical Fitness (mean ± sd)	
20 m Sprint (sec)	3.68 ± 0.56
*T*-test (sec)	13.05 ± 1.64
CMJ (cm)	26.55 ± 7.79
SQUAT (cm)	22.481 ± 6.87
YO-YO IR1 (laps)	7.22 ± 3.48
Handgrip (kg)	26.17 ± 6.60

^1^ BMI—Body Mass Index.

**Table 2:** Body composition indicators by sports (mean ± standard deviation).

	H (*n* = 35)	B (*n* = 25)	F (*n* = 42)	V (*n* = 22)	χ^2^/F	*p*	Post Hoc
Age (years)	12.53 ± 1.74	13.11 ± 1.70	14.39± 1.77	14.69± 1.68	10.865	<0.001	V > F > H
Occurrence of Menarche (yes %)	51.5%	64.0%	90.5%	100%	25.191	<0.001	V > F > B > H
Sports practice (years)	1.77 ± 1.19	3.76 ± 2.32	2.22 ± 2.59	1.26 ± 0.56	7.53	<0.001	B > F > H > V
Height (cm)	152.27 ± 10.18	156.52 ± 10.31	157.39 ± 7.93	159.95 ± 7.28	7.724	0.013	V > H
Weight (kg)	46.88 ± 12.92	54.72 ± 15.61	53.79 ± 10.63	53.04 ± 8.35	2.844	0.041	B > H
BMI (kg/m^2^)	19.92 ± 4.09	21.98 ± 4.57	21.71 ± 3.95	20.73 ± 2.83	1.838	0.144	-------
%BF (%)	22.44 ± 7.93	26.17 ± 8.52	24.78 ± 7.96	22.51 ± 6.11	1.526	0.211	-------
Fat-Free Mass (kg)	35.49 ± 7.00	39.32 ± 7.87	39.83 ± 4.83	40.71 ± 4.48	4.494	0.005	F > H; V > H

Legend: H—Handball; B—Basketball; F—Football; V—Volleyball; BMI—Body Mass Index; %BF—% Body Fat.

**Table 3:** Effects of different sports on body composition, controlling age, menarche, and time of sports practice.

	Age and Menarche Control			Age, Menarche, and Sports Practice Control		
	F	*p*	Post Hoc	F	*p*	Post Hoc
Height (cm)	0.830	0.480	---------	0.469	0.705	---------
Weight (kg)	2.369	0.074	---------	1.701	0.171	---------
BMI (kg/m^2^)	1.835	0.145	---------	1.825	0.146	---------
%BF (%)	2.269	0.084	---------	2.042	0.112	---------
Fat-Free Mass (Kg)	1.928	0.129	---------	1.000	0.395	---------

Legend: BMI—Body Mass Index; %BF—% Body Fat.

**Table 4:** Physical fitness tests by sports (mean ± standard deviation).

	H	B	F	V	F	*p*	Post Hoc
20 m Sprint (s)	3.73 ± 0.74	3.64 ± 0.31	3.72 ± 0.30	3.58 ± 0.46	0.620	0.604	-------
*T*-test (s)	12.91 ± 2.59	12.38 ± 0.70	13.21 ± 1.41	13.32 ± 0.92	1.208	0.311	---------
CMJ (cm)	26.49 ± 5.59	26.54 ± 6.18	27.28 ± 5.45	31.97 ± 5.54	4.474	0.005	V > H; V > B; V > S
SJ (cm)	21.86 ± 4.74	21.77 ± 4.96	22.75 ± 4.79	28.40 ± 4.68	8.880	<0.001	V > H; V > B; V > S
YO-YO IR1 (laps)	6.48 ± 3.54	7.94 ± 3.93	7.37 ± 3.56	6.89 ± 2.90	0.439	0.726	---------
Handgrip Strength (kg)	25.16 ± 5.53	30.88 ± 4.36	27.46 ± 5.24	27.16 ± 4.13	4.198	0.008	B > H

Legend: H—Handball; B—Basketball; F—Football; V—Volleyball.

**Table 5:** Effects of different sports on physical fitness, controlling age, menarche, and time of sports practice.

	Age and Menarche Control			Age, Menarche, and Sports Practice Control		
	F	*p*	Post Hoc	F	*p*	Post Hoc
20 m Sprint (s)	0.894	0.447	---------	0.850	0.470	---------
*T*-test (s)	3.184	0.027	V > H	2.125	0.102	---------
CMJ (cm)	3.992	0.010	V > B; V > S	4.146	0.008	V > B; V > S
SJ (cm)	7.880	<0.001	V > B; V > S; V > H	7.686	<0.001	V > H; V > B; V > S
YO-YO IR1 (laps)	0.965	0.412	---------	0.409	0.747	---------
Handgrip strength (kg)	4.031	0.009	B > S; B > V	2.319	0.080	---------

## Data Availability

The data presented in this study are available on request from the corresponding author.

## Abbreviations

The following abbreviations are used in this manuscript:

CMJ	Countermovement jump
SJ	Squat jump
Yo-Yo IR1	Yo-Yo Intermittent Recovery Test Level 1
